# Appetite, reward, and obesity: the causes and consequences of eating behaviors

**DOI:** 10.3389/fpsyg.2015.00411

**Published:** 2015-04-09

**Authors:** Tanya Zilberter

**Affiliations:** Infotonic ConseilMarseille, France

**Keywords:** eating behavior, eating disorders, brain metabolism, obesity, reward, appetite, hedonic eating, energy expenditure

Eating behavior is constantly being shaped by a convergence of homeostatic demands and motivational parameters set to insure adequate behavioral patterns with a variety of metabolic consequences that send signals to brain structures to the brain structures responsible for the homeostatic parameters. Out of the scores of themes concerning eating behavior, the 18 papers comprising the current Research Topic, “*The two-way link between eating behavior and brain metabolism*,” happened to be distributed among the three large overlapping fields: reward, appetite, and obesity.

## Reward

Understanding eating behavior is impossible without understanding the mechanisms of reward. Blum et al. (2014) looked at obesity as a result of over-consumption due to addictive processes and impaired reward functioning. They discuss the relevancy of the Reward Deficiency Syndrome: a genetic/epigenetic phenomenon resulting in a dysfunction of the dopaminergic system intertwined with the system of carbohydrate homeostasis. Natural palatable reward is powerfully mediated by sensory input stimulated by carbohydrates. The specifics of peripheral stimulation (oral, duodenal, or combined sensing of sweet) addressing to the brain areas involved with hedonic processes allowed an important conclusion regarding the role of visceral modulation in hedonic processes (Clouard et al., 2014). The rewarding nature of food mediated via dopaminergic system can be enhanced under the condition of excessive stress triggering a vicious circle and leading to maladaptive responses including overconsumption of palatable food and obesity (Sominsky and Spencer, 2014). The dopaminergic neurons are under influence of insulin and leptin—both potent regulators of eating behavior (Khanh et al., 2014), thus adding an important loop to the eating behavior control system.

The interplay between reward, gratification, and dopaminergic system can override hunger control. This was the main theme of Singh's review (Singh, 2014) summarizing the links between three domains: mood, food, and obesity. Godier and Park (2014) discussed the role of modulation of the glutaminergic and gamma-aminobutyric acid (GABA) pathways by dopaminergic system in functioning of the reward system and suggested a potential application of these mechanisms to treatment of eating disorders, compulsivity, and addictions. Dietrich et al. (2014) linked heightened or reduced reward sensitivity and concluded that this link plays one of the key roles in the development of obesity. They also showed that the link between body weight status and behavioral characteristics are gender-specific. Interestingly, Pendergast et al. (2014) demonstrated that the source of reward may be unrelated to eating behavior: an access to a running wheel in obese mice, being a source of not only exercise but also a reward, normalized circadian rhythms of eating behavior disturbed due to the high-fat, high-sugar obesogenic diet.

## Appetite

Watkins and Kim (2015) investigated the role of endocannabinoid system in macronutrient metabolism and discussed the possible appetite-stimulating role of polyunsaturated fatty acids, which are precursor ligands of cannabinoid receptors. The appetite-inhibiting effects of brain-derived neurotrophic factor midbrain dopaminergic system, hedonic eating, reward and addiction has been thoroughly reviewed by Takei et al. (2014). Basing on their original data, the authors suggested that these activities are mediated by Mammalian Target of Rapamycin, the role of which authors defined as “*a cellular crossroads for the regulation of food intake and metabolism by nutrients*” (p. 4).

The effect of the ketogenic diet on the appetite-inhibiting brain network recruiting Cholecystokinin, neuropeptide Y and Ghrelin as well as on an appetite-stimulating network involving adiponectin, GABA and adenosine monophosphate-activated protein kinase is pictured in details in the review of the EB-related effects of the ketogenic diet by Paoli et al. (2015). In a mini-review, McFadden et al. (2014) analyzed the rope of alpha-7 nicotinic acetylcholine receptor in eating behavior and its interaction with proopiomelanocortin, neuropeptide Y, GABA, serotonin, glutamate, melanin-concentrating hormone, and dopamine.

One of the feasible means of appetite control might be a generic meal replacement: in a sample of older, obese adults, it affected brain areas relevant to eating behavior where it lowered functional connectivity in insula, anterior cingulate cortex, superior temporal pole, amygdala, and hippocampus (Paolini et al., 2014). The study may have practical application to the obesity management.

## Obesity

In agreement with the numerous data on the obesogenic diet, the combination of fat and sugars in snack foods triggered more profound overeating response compared with either fat or sugars or standard chow (Hoch et al., 2014). One of the most common consequences of obesity is hypertension. Smith et al. (2014) investigated the opposite link and found that an antihypertensive vasodilatory drug Losartan prevented obesity of rats fed on the obesogenic diet, perhaps via an increase in energy expenditure due to thermal dissipation through the skin.

Diet-induced obesity, human binge eating behavior and the mesolimbic pathway were analyzed by Perello et al. (2014). They discussed the integration of neuronal inputs from the hypothalamus with peripheral hormones and visceral sensory information in the arcuate hypothalamic neurons. Messina et al. (2014) reviewed the role of hypothalamic neurons, both excited and inhibited by glucose, and their interaction with Orexin-A in coordination of such processes as feeding, sleep-wakefulness, neuroendocrine function, vascular, and metabolic reactions. In the review discussing the obesity-depression relationship, Rossetti et al. (2014) described the coexisting pathways for energy homeostasis and mood balance and suggested that obesity might be considered a risk factor for depression but most likely it happens in the cases of either binge eating or metabolically precarious, abdominal adiposity.

Orexin-A can cause both hyperphagia and hypophagia; it can also modify energy expenditure through thermal dissipation (Messina et al., 2014). This is an important observation since the energy expenditure aspect is often overlooked in the obesity studies. Adding the possibility to voluntarily enhance energy expenditure eliminated the obesogenic diet's effect of high-fat, high-sucrose diet (Pendergast et al., 2014).

## Conclusion

The Topic collected and connected information concerning both the underlying metabolic mechanisms and consequences of eating behaviors - the aspects tremendously important for a better understanding of normal and pathological eating behavior to better manage appetite and obesity as well as for improving professional and public education on eating and metabolic disorders.

### Conflict of interest statement

The author declares that the research was conducted in the absence of any commercial or financial relationships that could be construed as a potential conflict of interest.
